# Fellow-Workers and the Roll of Honour

**Published:** 1915-10-02

**Authors:** 


### ROUND THE WORLD.

[The Editor invites Early Information and Contributions Marked "Fellow-Workers."]

### Fellow-Workers and the Roll of Honour.

Dr. Hamilton Clelland Marr, one of the Scottish Lunacy Commissioners, whose work in connection with the nerve and mental cases amongst the wounded at the Royal Victoria Hospital, Edinburgh, was recently referred to in these notes (The Hospital, August 7, 1915), has now been appointed specialist for nervous diseases with the troops in the Mediterranean. On taking up his new duties Dr. Marr has been given military rank as a major in the R.A.M.C.

Major James Mowat, R.A.M.C., whose life has been lost in the Near East, was one of the four medical officers who went down with the *Royal Edward*. It was a sad end to a very remarkable career, for the late officer had not only completed twenty years' service with the Royal Navy, and retired with the rank of fleet-surgeon some years before the outbreak of war, but, having joined the Navy again at the outset of hostilities, he was on the *Hermes* when that ship was torpedoed last year, after which experience he had to resign his commission on account of illness. Nevertheless he recovered his health, and on offering himself to the War Office was appointed major with ambulance duties. A student of Aberdeen, where he graduated in 1891, Major Mowat thus had the unique experience of having had two bouts of responsible service with the Royal Navy, a spell of private practice, and an important commission in the R.A.M.C.

The M.O.H. for Leamington, Dr. Herbert Gibbons Ward, has joined the long list of medical men in public health service who have left for military work. An M.D., Ch.B. of Manchester University, he was formerly junior assistant and junior research Fellow in the Manchester Public Health Laboratory, and assistant M.O.H. for Derby. It is understood that during Dr. Ward's absence his duties will be divided between Dr. C. E. Tangye, M.O.H. for Mid-Warwickshire, and Dr. Miles H. C. Atkinson, of Leamington.

Amongst medical officers who have been killed or have died of wounds must now be included Lieutenant-Colonel C. E. Thomas, V.D., Major J. L. Duval, R.A.M.C., Lieutenant P. T. Warren, R.A.M.C. (T.F.), Lieutenant T. A. Peel, R.A.M.C., and Lieutenant C. M. Harris, R.A.M.C.

Lieutenant-Colonel Charles Ernest Thomas, V.D., was serving with the New Zealand Expeditionary Corps at the time of his death. A Middlesex Hospital student, he qualified by taking the diplomas of L.S.A. and L.R.C.S. Edin. in 1888, and subsequently settled in practice in New Zealand, holding various public appointments. Lieutenant-Colonel Thomas was an old campaigner, having served with distinction during the South African War, when he worked with the New Zealand contingent.

Major J. L. Duval was attached to the Canadian Army Medical Corps. On the outbreak of war he joined one of the Canadian Field Ambulances, being given a commission as captain, and after some six months' keen work being promoted to major.

Lieutenant Peyton Tollemache Warren, R.A.M.C. (T.F.), was also a Dublin student, but some five years senior to the last-named in respect of qualification. Settling down in Wales, he became medical officer and public vaccinator for Bryn, Port Talbot, Glamorganshire, and a well-known leader of local sporting movements. Lieutenant Warren had joined a Welsh Field Ambulance as medical officer two years before the call came for active service.

Lieutenant Thomas Alfred Peel, R.A.M.C., was yet another Irish student; but instead of qualifying in his native land he proceeded to Newcastle-on-Tyne for his final studies, and graduated M.B., B.S. of Durham University in 1911. A good all-round man, who displayed considerable literary as well as scientific ability, Lieutenant Peel had held resident appointments at the Royal Gwent Hospital, Newport, the Stafford General Infirmary, and the North Stafford Infirmary at Stoke.

It is announced that the following gentlemen will form the staff of the Anglo-Russian Hospital which is to work in Russia: Dr. A. M. Fleming, C.M.G. (commandant and chief sanitary officer), Mr. H. F. Waterhouse (chief surgeon), Dr. T. J. Horder (physician), with Dr. Mark Gardener, Mr. G. A. Jones, Dr. G. May, Mr. A. B. Rosher, and Mr. H. F. Q. Thompson as assistant medical and surgical officers.

Mr. Herbert Furnival Waterhouse, M.D. Edin., F.R.C.S. Eng., is surgeon and lecturer on surgery at Charing Cross Hospital, senior surgeon to the Victoria Hospital for Children, and consulting surgeon to the Kensington Children's Hospital. Graduating at Edinburgh in 1887, after distinguishing himself as a student, this well-known surgeon held various resident appointments in Scotland.

Major Cuthbert Sidney Wallace, R.A.M.C., surgeon to St. Thomas's Hospital, has been working at the British Red Cross Hospital at Netley, where he has given special attention to the uses of certain forms of splinting in the treatment of gunshot wounds of the thigh. Assisted by Captain Maybury, R.A.M.C., he has published an interesting report (the *Lancet*, September 4, 1915), showing the advantages of the methods in question. Major Wallace, who qualified in 1891, subsequently became an F.R.C.S. Eng., and won the gold medal in obstetric medicine at the M.B. Lond., whilst qualifying for the gold medal at the B.S. Lond. He was at one time surgeon to the East London Hospital for Children.

Captain Bernard Constable Maybury, M.B., B.S. Lond., F.R.C.S. Eng., is really resident assistant surgeon to St. Thomas's Hospital, but his military duties have taken him to Netley. Qualifying in 1910, he had a brilliant career as a student, winning the Musgrove scholarship, the Cheselden medal, and the Treasurers' gold medal at St. Thomas's.

In the notes referring to the work of the late Lieutenant-Colonel Dauber, who lost his life in the *Royal Edward* disaster, it should have been mentioned that his registrarship at the Soho Hospital for Women was followed by many years' useful service as assistant surgeon, during which he did a great deal for the institution. He had just become full surgeon when his military duties called him away.

